# Clinical indications, image acquisition and data interpretation for white blood cells and anti-granulocyte monoclonal antibody scintigraphy: an EANM procedural guideline

**DOI:** 10.1007/s00259-018-4052-x

**Published:** 2018-05-31

**Authors:** A. Signore, F. Jamar, O. Israel, J. Buscombe, J. Martin-Comin, E. Lazzeri

**Affiliations:** 1 Nuclear Medicine Unit, Department of Medical-Surgical Sciences and Translational Medicine, “Sapienza” University of Rome, Ospedale S. Andrea, Via di Grottarossa 1035, 00189 Rome, Italy; 2 Department of Nuclear Medicine, Université Catholique de Louvain, Brussels, Belgium; 3 Department of Nuclear Medicine, Rambam Health Care Campus, Haifa, Israel; 4 Department of Nuclear Medicine, Cambridge Biomedical Campus, Cambridge, UK; 5 Nuclear Medicine Department, Hospital Universitario de Bellvitge, Barcelona, Spain; 6 Regional Center of Nuclear Medicine, Azienda Ospedaliero-Universitaria Pisana, Pisa, Italy

**Keywords:** Infection, Inflammation, Acquisition protocols, WBC, Monoclonal antibodies

## Abstract

**Introduction:**

Radiolabelled autologous white blood cells (WBC) scintigraphy is being standardized all over the world to ensure high quality, specificity and reproducibility. Similarly, in many European countries radiolabelled anti-granulocyte antibodies (anti-G-mAb) are used instead of WBC with high diagnostic accuracy. The EANM Inflammation & Infection Committee is deeply involved in this process of standardization as a primary goal of the group.

**Aim:**

The main aim of this guideline is to support and promote good clinical practice despite the complex environment of a national health care system with its ethical, economic and legal aspects that must also be taken into consideration.

**Method:**

After the standardization of the WBC labelling procedure (already published), a group of experts from the EANM Infection & Inflammation Committee developed and validated these guidelines based on published evidences.

**Results:**

Here we describe image acquisition protocols, image display procedures and image analyses as well as image interpretation criteria for the use of radiolabelled WBC and monoclonal antigranulocyte antibodies. Clinical application for WBC and anti-G-mAb scintigraphy is also described.

**Conclusions:**

These guidelines should be applied by all nuclear medicine centers in favor of a highly reproducible standardized practice.

## Introduction

Scintigraphy using radiolabelled white blood cells (WBC) and anti-granulocyte monoclonal antibodies (anti-G mAb) has been used in a variety of different clinical situations [1, 2]. While it is difficult to draw evidence-based statistical conclusions for some clinical applications, for other infectious processes the role of these imaging tools is well standardized, with grade A or B levels of evidence.

Here we summarize the most important clinical indications in which WBC or anti-granulocyte mAbs can be used and the way images should be acquired and interpreted. It is not the aim of this guideline to compare the indication of different nuclear medicine modalities and radiopharmaceuticals in clinical practice. Several reviews are available for advanced and more comprehensive readings on the topic, including four papers which have been recently published with meta-analysis of data collected between 1985 and 2005 on the clinical use of radiolabelled WBC as compared to other available diagnostic techniques [3–6].

The main aim of these guidelines is to support and promote good clinical practice. Nevertheless, guidelines are used in the complex environment of a health care system with its ethical, economic, legal and other aspects that need to be taken into consideration in each country.

### Autologous WBC and anti-G-mAbs

Autologous white blood cells (mainly neutrophils) can be radiolabelled ex-vivo using ^99m^Tc-HMPAO or ^111^In-oxine as described elsewhere [7, 8] using sterile conditions in accordance with national regulation on the production of medicines. The recent availability of disposable sterile closed devices for WBC labelling (Leukokit® and WBC Marker kit®) [9], functioning as disposable mini-isolators, has enormously facilitated the labelling procedure that, however, remains time consuming and requires withdrawing 30–40 ml of blood from patient. Once injected i.v. in the patient, radiolabelled WBC migrate rapidly to the lungs and, if not damaged, proceed to liver, spleen and the reticulo-endothelial system, including bone marrow. Approximately 1 h after injection, labeled cells further migrate to bone marrow and, in case of an infection, to the infected tissue due to chemotactic attraction caused by biofilm and its soluble products. The rate of accumulation of labeled cells in infection sites depends on the site of infection (cardio-vascular tissue may accumulate WBC within a few hours and much more rapidly than peripheral bones and CNS), on the virulence and extent of infection (some bacteria produce more chemotactic factors than others and some chronic infections or abscesses may show minimal accumulation of leukocytes at late time points), on the type of pathogen (fungal infection may attract less WBC than bacterial infections), on the assumption of antibiotic or steroid therapy (that may reduce bacterial virulence or leukocyte migration) and on the status of vascularization of the infected tissue (capillaries in the vertebral body may be compressed by oedema in case of spondylitis thus preventing WBC accumulation as well as capillaries in diabetic patients with micro-macro angiopathy/neuropathy or in patients with severe atherosclerotic lesions).

With the aim of simplifying the procedure of WBC labelling, some anti-granulocyte monoclonal antibodies have been described. Two products are commercially available: a whole murine IgG anti-NCA-95 antibody (Besilesomab, Scintimun®) [10], and a Fab’ fragment anti-NCA-90 (Sulesomab, Leukoscan®) [11]. Both radiopharmaceuticals bind to peripheral neutrophils but with substantial biodistribution differences [12]. Besilesomab accumulates more than Sulesomab in normal bone marrow, but binds more efficiently to neutrophils in blood and at sites of infection. It has lower plasma disappearance than Sulesomab, thus mimicking the biodistribution of radiolabelled autologous WBC. It is also more stable in plasma, and images at 20–24 h after injection are more reliable than if using Sulesomab. Furthermore, Besilesomab, being a murine antibody may induce production of human anti-mouse antibodies (HAMA) that must be checked before performing the study and limit its use to one single administration in life. Sulesomab, being a Fab’ fragment, does not induce HAMA production and can be re-used several times in the same patient, but it is licensed in Europe only for peripheral musculoskeletal infections.

#### Osteomyelitis and spondylodiscitis

Osteomyelitis (OM) is an infection of the bone and bone marrow due to the presence of aerobic or anaerobic micro-organisms, viruses and/or fungi. OM can represent a complication of a systemic infection or can be an infectious process located primarily in the bone or surrounding tissue. Of particular clinical relevance is the differential diagnosis between OM and soft tissue infection in diabetic patients with Charcot foot or forefoot infections [13–15].

The most frequent origin of primary OM is haematogenic but the micro-organisms can also reach the site of infection directly (in patients with exposed fractures or following surgical procedures, as in sternal infections) or simply *per continuitatem*. The haematogenic OM is often caused by gram positive micro-organisms, whereas fungi and mycobacteria frequently produce direct and chronic infections. The conditions that predispose to primary OM include immunosuppression, previous radiotherapy, diabetes mellitus (diabetic foot), drug addiction and hematological chronic disorders (e.g. sickle-cell anemia). Secondary OM is most often associated with (open) trauma, surgery and especially prosthetic implants (see separate paragraph).

OM can be classified as acute, subacute and chronic according to the type of onset and clinical course. Patients with acute OM usually present with fever, leukocytosis, elevated acute phase reactants (ESR and CRP) and mild or intense pain in the affected region. Redness, pain, swelling and impaired function (the classic rubor, tumor, dolor, calor, function laesa as described by Galen) may also be present.

The diagnosis of OM can be challenging. Radiological techniques, such as computed tomography (CT) and magnetic resonance imaging (MRI) play an important role, complemented by radionuclide imaging procedures (mainly radiolabelled white blood cells with nanocolloid scan for bone marrow imaging, but also anti-G-mAb scintigraphy) with an overall accuracy that exceeds 90% [12, 16–22].

Radiolabelled WBC is not a valid radiopharmaceutical for the diagnosis of vertebral infections, the frequent lack of uptake in the site of infection, as well as in other benign spine pathology, does not allow for accurate diagnosis or useful follow-up. [^18^F]FDG PET/CT in association with MRI provides the highest diagnostic accuracy [23–26]. Nevertheless, in early spondylodiscitis (within 1 week from onset of symptoms) and in para-vertebral soft tissue infections associated with spondylodiscitis, WBC scan can be used.

Clinical indications for nuclear medicine evaluation:

- Peripheral bone osteomyelitis (OM)
- Suspected OM
- Evaluation of extent of OM
- Evaluation of treatment response
- Spondylodiscitis
- Para-vertebral soft tissue infection in spondylodiscitis

#### Joint prosthesis and other orthopedic hardware

More than a million hip replacements are performed each year worldwide, and the number of other artificial joints (shoulder, elbow, hip, knee) inserted is also rising. With increasing numbers of implantations, mechanical and infected loosening of the prostheses have become more common. The risk of infection is highest during the first two years after implantation. Differentiating infection from aseptic loosening is difficult because the clinical presentation and the histopathological changes in both entities can be similar, but at the same time is extremely important because the treatment of these two entities is different. Joint aspiration with gram stain and culture is considered the definitive diagnostic test; its sensitivity, however, is variable, ranging from 28 to 92%; specificity is more consistent, ranging from 92 to 100%. Plain radiographs are neither sensitive nor specific and cross-sectional imaging modalities (CT and MRI) are limited by hardware induced artifacts. Radionuclide imaging (both WBC and anti-G-mAb scintigraphy) that is often not affected by metallic hardware can play an important role in the diagnosis of prosthetic joint infection. The evaluation of hybrid images (SPECT/CT) can be also performed after imaging reconstruction without attenuation correction, with an overall accuracy ranging from 88 to 98% (being highest for WBC combined with bone marrow imaging) [21, 27–30].

Clinical indications for nuclear medicine evaluation:

- Suspected septic loosening
- Evaluation of extent of infection
- Evaluation of treatment response
- Suspected infective post-traumatic pseudo-arthrosis
- Exclusion of infection in patients with antibiotic spacer before prosthesis re-implant

#### Inflammatory bowel diseases

Inflammatory bowel diseases (IBD) mainly include Crohn’s Disease (CD) and Ulcerative Colitis (UC). Ultrasound (US), CT and MRI are of particular use in evaluating the presence of extramural complications such as abscesses, fistula and perforations [31]. These techniques therefore usually are the first choice imaging methods due to their availability and good accuracy. WBC scintigraphy allows additional information to be obtained regarding the degree of activity of disease and its extent [31, 32]. There are some limitations to labeled WBC imaging in IBD. The test cannot identify anatomical changes such as strictures, which are best delineated with endoscopy and contrast radiography. It is less sensitive for disease affecting the upper tract than the lower gastrointestinal tract and may also be affected by steroid administration [32, 33].

Clinical indications for nuclear medicine evaluation:

- Evaluation of activity of disease
- Evaluation of extent of disease
- Differential diagnosis between inflammatory and fibrotic strictures
- Early assessment of disease relapse after surgery
- Evaluation of treatment response

#### Fever of unknown origin

Fever of unknown origin (FUO) is defined as a body temperature higher than 38.3 °C persisting for two weeks or more, remaining unexplained after 3-day inpatient or two-week outpatient observation. Beside the classical ‘pure’ FUO, Durack identified related conditions encompassing neutropenic FUO, HIV-associated immune depression FUO and nosocomial FUO [34]. Diagnosis is often made by a good anamnestic assessment with special attention to occupational and recreational exposure to pathogens, a recent travel history or drug abuse. Laboratory tests are also relevant but may be very unspecific and radiological imaging may be helpful although in most cases the origin of FUO remains uncertain [34].

Scintigraphy with labeled WBC and PET with [^18^F]FDG are additional imaging modalities to evaluate patients with FUO with high sensitivity, thus a negative study virtually excludes an infection as the cause of the fever [35–38]. It is accepted that when patients with FUO have low probability of infection (low ESR, WBC count and CRP) the first scan to be performed should be [^18^F]FDG. If patients have a high clinical probability for infection they should first perform a WBC scan. There are also reports on the use of radiolabelled anti-granulocyte mAb in FUO with comparable diagnostic accuracy as for WBC.

Clinical indications for nuclear medicine evaluation:

- Evaluation of unknown site of infection in patients with high pre-test probability of infection
- Evaluation of extent of disease

#### Soft-tissue infections

##### Postoperative infections

Nuclear medicine procedures can be a useful adjunct to morphologic imaging and can facilitate the differentiation of abscesses from other fluid collections, tumor, and normal postoperative changes. Labeled WBCs rarely accumulate in uninfected tumors and do not, with few exceptions, accumulate in normally healing surgical incisions [39–41]. Consequently, because of this high specificity, WBC imaging is the preferred radionuclide test for the evaluation of postoperative infections [42].

##### Cardiovascular system infections

Echocardiography is readily available and accurately diagnoses bacterial endocarditis. Nevertheless, the use of radiolabelled WBC scintigraphy accurately detects infective endocarditis and extra-cardiac septic embolisms [43–47]. Prosthetic vascular graft infections are also first studied by angio-CT, and labeled WBC imaging is a useful complement [48–52]. The accuracy of WBC imaging for diagnosing prosthetic vascular graft infection is above 90%, and the use of [^18^F]FDG is also a highly accurate alternative [53]. No role of WBC scan has been suggested for imaging vasculitis.

Clinical indications for nuclear medicine evaluation:

- Infective endocarditis (IE)
- Suspected IE with doubtful ultrasound
- Evaluation of septic embolism in certain IE
- Evaluation of treatment response
- Differential diagnosis with marantic vegetations
- Vascular graft infections
- Diagnosis of infection
- Evaluation of extent of disease
- Evaluation of disease activity
- Evaluation of treatment response

##### Pulmonary infections

Although pulmonary uptake of labeled WBC is a normal event during the first few hours after injection, its appearance after 24 h is abnormal.

Diffuse pulmonary activity on images obtained more than 4 h after injection of labeled WBC can be due to opportunistic infections, radiation pneumonitis, pulmonary drug toxicity and adult respiratory distress syndrome. A diffuse pulmonary uptake pattern is also seen in septic patients with normal chest radiographs who have no clinical evidence of respiratory tract inflammation or infection. It is important to note that while diffuse pulmonary activity on labeled WBC images is associated with numerous conditions, it has been only very rarely described in bacterial pneumonia [54]. Focal pulmonary uptake that is segmental or lobar is usually associated with bacterial pneumonia. This pattern can be also seen in patients with cystic fibrosis and is due to WBC accumulation in pooled secretions in bronchiectasic regions. Non-segmental focal pulmonary uptake is caused by technical artifacts caused by faulty labelling or reinjection and is usually not associated with infection and disappears within 4 h post injection.

Clinical indications for nuclear medicine evaluation:

- Diagnosis of bacterial pneumonia
- Differential diagnosis of infective and neoplastic lesions
- Evaluation of treatment response

##### Central nervous system infections

The differential diagnosis of a contrast enhancing brain lesion identified on CT or MRI includes abscess, tumor, cerebrovascular accident, and even multiple sclerosis. WBC scintigraphy provides valuable information in the differential diagnosis of contrast enhancing brain lesions [55–61] and in the follow-up of patients with malignant otitis. False negative results have been reported in patients receiving high doses of steroids [61]. The use of anti-G-mAb can also be useful in these situations [62, 63].

Clinical indications for nuclear medicine evaluation:

- Cerebral hypodensity CT lesions with hypervascularized peripheral ring
- Differential diagnosis of cerebral lesions in HIV patients

#### Other applications of radiolabelled WBC and mAbs

##### Aids

WBC scintigraphy plays a limited role in the evaluation of AIDS-related infection. The test is not as sensitive as CT for detecting opportunistic infections of the lungs and lymph nodes. The test is useful, however, for detecting colonic infections in the HIV(+) patient [64–66]. It must be kept in mind that handling blood from HIV-infected patients carries a risk for both the staff and fellow patients if multiple labelling procedures are performed at the same time, and recommendations regarding this specific issue have been previously published [1, 67, 68].

### Image acquisition protocols

The use of anti-G-mAb and WBC scintigraphy has many useful fields of application. Nevertheless, the image acquisition protocols are not always identical, and in addition, there may be some variation in the criteria used in image interpretation. It is therefore important to standardize, based on published evidence and on the experience of the authors, the acquisition and interpretation protocols for each disease. Therefore, the aim of this guideline is to provide general recommendations for improving clinical practice and to minimize the occurrence of artifacts and interpretation hurdles.

For all studies using labeled blood products, it is essential that measures are in place for correct patient identification in order to avoid cross-administration of labeled cells to the wrong patient [7, 8].

The potential interference of some drugs and antibiotics with cell labelling has to be considered [67, 68]. However, patients receiving antibiotic treatment should not be excluded “a priori” since reports regarding their effect on WBC scintigraphy give various results. While several authors describe no interference between such treatment and the accuracy of WBC/mAbs scanning, others advise delaying the scan for at least 1–2 weeks after therapy withdrawal or, in the case of doubtful study results in patients receiving antibiotics, to repeat the test 2 weeks later. The decision whether to perform or cancel the study depends entirely on the clinical setting and must be discussed case-by-case with the referring physician.

#### Acquisition protocols for ^99m^Tc-labeled WBC and anti-G-mAb scintigraphy

A large-field-of-view gamma camera with a low-energy high-resolution collimator should be used. Camera resolution should always be set to zoom 1 at 512 × 512 pixels with a high resolution collimator.

For ^99m^Tc labeled WBC studies, an in vivo quality control should be performed including imaging of lungs, liver and spleen at 30 min post-injection (p.i.) (early images). Normal bowel activity is seen in 20–30% of children at 1 h and 2–6% of adults at 3–4 h p.i. According to the clinical indication, whole-body, planar and, if appropriate, SPECT (or SPECT/CT) images should be performed at 3–4 h (delayed images) and 20–24 h (late images).

For ^99m^Tc labeled anti-granulocyte mAbs scintigraphy early lung images are not necessary but (as applicable) planar, SPECT (or SPECT/CT) and whole-body images at 3–4 h and 20–24 h should be performed.

According to different biodistribution of labeled WBC in blood, bone-marrow, infection and sterile inflammation, three sets of images must be generally acquired of the region of interest: “early images” (within 30 min and 1 h p.i.), “delayed images” (between 2 h and 4 h p.i.) and “late images” (between 20 h and 24 h p.i.). Early images provide useful information on the lung transit, on liver/spleen ratio (which should be at least 1:2 but may also depend on the neutrophil:eosinophil ratio since neutrophils preferentially migrate in the liver and eosinophils; that uptake HMPAO much more avidly than neutrophils, preferentially migrate in the spleen) on bone marrow distribution and on vascular pattern. Sites of infection should be visible on delayed images with further accumulation of labeled leukocytes seen in the late images due to increased uptake in infected areas and reduction in background activity. Over time there is also reduction or stable activity in bone marrow and non-specific activity in the bowel due to Tc-HMPAO excretion from the liver.

Several acquisition protocols have been suggested for both ^99m^Tc-labeled WBC and anti-G mAb scintigraphy. Fixed counts per image or fixed times per image at all time points are the most commonly used protocols but also the most difficult to be interpreted because of interference of radioactivity from other organs and operator bias in image display. For fixed counts or time acquisitions, images of the regions of interest are acquired for at least 500,000 counts or 5–10 min with a large field of view including a region of normal bone marrow as a reference (e.g. iliac bone, sternum or skull).

These methods, and particularly the acquisition of several images at several time-points with the same amount of counts, is discouraged or recommended for expert readers.

In order to reduce operator dependence in image display and final interpretation, we recommend acquiring images time-corrected for isotope decay (see Table 1). If early images are acquired with a set number of counts or time, delayed and late images should be corrected for the isotope half-life. With this acquisition modality, different images can be compared with the same intensity scale, in absolute counts (and not in % of maximum activity) thus avoiding any operator bias [1]. This method also allows to correctly detect a true increase of uptake in suspected regions over time, an important criteria of positivity for bone infections and for most soft tissue infections. The method has been recently validated in a multicentre European study [69, 70] as being highly accurate for osteomyelitis, prosthetic joint infections, diabetic foot osteomyelitis and some soft tissue infections (such as dermal filler infections, endocarditis, abscesses, brain infections and sternal wound infections) but not for soft tissue infection in diabetic foot [14, 71].

**Table 1:** How to calculate acquisition time according to decay of ^99m^Tc (A) and ^111^In (B)

A						
Hours^a^	Acquisition time (seconds) for ^99m^Tc decay corrected images				exp(-λt)	λt
*0* *(Early images)*	100	150	200	300		
*1*	112	168	224	337	0.8909	0.1155
*2*	126	189	252	378	0.7937	0.231
*3*	141	212	283	424	0.7072	0.3465
*4*	159	238	317	476	0.6300	0.462
*6*	200	300	400	600	0.5001	0.693
*8*	252	378	504	756	0.3969	0.924
*14*	504	756	1008	1511	0.1985	1.617
*20*	1007	1511	2015	3022	0.0993	2.31
*22*	1269	1904	2538	3808	0.0788	2.541
B						
Hours^a^	Acquisition time (seconds) for ^111^In decay corrected images				exp(-λt)	λt
*0* *(Early images)*	100	200	300	400		
*1*	101	202	303	404	0.9897	0.0103
*2*	102	204	306	408	0.9795	0.0206
*3*	103	206	309	413	0.9694	0.0310
*4*	104	208	313	417	0.9595	0.04137
*6*	106	213	319	426	0.9398	0.06205
*8*	109	217	326	435	0.9206	0.08274
*14*	118	236	354	472	0.8475	0.16549
*20*	123	246	369	492	0.8131	0.20686
*22*	125	250	374	499	0.7965	0.22755

^a^Time *0* is the time of the first scan not necessarily the time of injection

SPECT or SPECT/CT is useful in most types of infection but should be considered mandatory in a few selected indications (e.g. endocarditis, diabetic foot, vascular prosthesis). If SPECT or SPECT/CT is used for quantitative purposes, this should be acquired in a 128 × 128 matrix utilizing the same decay-corrected protocol as described above (i.e. 5 h post-injection for 7 s/step and 20 h post-injection for 40 s/step, because of 15 h difference between the two scans). If acquired with a decay-corrected protocol, SPECT images can also be used for semi-quantitative purposes to evaluate any increase of T/B ratio with time.

Typically, however, a SPECT/CT is performed after planar images (4 or 5 h post injection) only to provide the best anatomical localization of WBC accumulation, but the positivity for infection is given by comparing delayed and late planar images. Therefore, the SPECT/CT is not required for semi-quantitative purposes and acquisition at 4–5 h p.i. can be acquired with 20–30 s/step (depending on the injected activity) and acquisitions at 20–24 h p.i. are often not necessary (even because would require a very long acquisition time) but can be also performed if new sites of pathological uptake appear that were not detected at 4–5 h scan (in this case acquisition time should be at least 30–50 s/step, also depending on the injected activity and on the region to be imaged, with time longer for peripheral parts and shorter for the abdomen).

When using ^99m^Tc-HMPAO-labeled WBC for abdominal infections and IBD, images should only be acquired at 30 min and 2–3 h after injection of the labeled WBCs. This is because a metabolite of ^99m^Tc-HMPAO is released by WBC with time, taken up by the liver and excreted via the bowel, thus producing false positive images at later time points. Only in cases with a suspected fistula or abscess it is necessary to acquire images at a later time point, at 4–6 h after radiopharmaceutical injection (or even 20–24 h post injection). This pitfall does not occur with ^111^In-labeled WBC that are therefore preferable for studying abdominal infections. For the same reason, when using ^99m^Tc-labeled WBC, vascular graft infections of abdominal vessels (aorto-bi-iliac grafts) should be imaged within 3 h from administration of labeled cells. A dynamic acquisition (one image every 5 s for the first 150 s after injection) may help to map the vascular structures and detect obstructions or aneurisms. For all these intra-abdominal and pelvic infections, SPECT/CT can be extremely helpful (if not mandatory), allowing for a precise localization of an infectious focus to the vascular graft or adjacent soft tissues only.

With anti-G-mAbs, image protocols differ between complete and fragmented antibodies: images with complete ^99m^Tc-anti-NCA-95 antibody should be performed at 2–4 h p.i. and 16–24 h p.i. in planar whole body technique because a significant increase in sensitivity and specificity will be achieved with delayed 24 h images due to higher target to background ratios (T/B). Planar images can be performed with an acquisition protocol time-corrected for isotope decay, as mentioned above for WBC. The best time point for SPECT images is 4–6 h after injection but another SPECT at 16–24 h p.i can also be performed if required, similarly to WBC scan.

With ^99m^Tc-anti-NCA-90 (Fab’) antibodies, images should be performed 1 h p.i. and 4–6 h p.i. in whole body as well as in single scan technique. Acquisition protocols are also better using time-corrected for isotope decay. SPECT of suspected central bone infection should be performed at 4–6 h p.i., whereas in case of suspected endocarditis delayed SPECT images at 16–24 h p.i. have also been suggested [45]. The combination of ^99m^Tc-anti-NCA-90 (Fab’) antibodies and ^99m^Tc-HDP three phase bone scan can be useful to rule out false positive accumulation of the antibody fragment due to non-specific inflammatory oedema. Others have suggested also performing late images 20–24 h after injection to improve the specificity [72, 73], although this protocol and the specificity of ^99m^Tc-anti-NCA-90 (Fab’) antibodies have been criticized by others [74, 75].

For both antibodies, as for labeled WBC, SPECT/CT should be considered mandatory for endocarditis, diabetic foot and vascular prosthesis.

#### Acquisition protocols for ^111^In-labeled WBC scintigraphy

A large-field-of-view gamma camera with a medium energy collimator should be used. Both ^111^In photopeaks should be acquired at 173 and 245 keV (±10%). If using a time-saving acquisition protocol with both ^99m^Tc-colloid for bone marrow imaging performed in orthopedic cases prior to ^111^In-WBC re-injection, only the upper peak of ^111^In should be subsequently acquired (245 keV) to avoid cross-contamination of ^99m^Tc into the lower peak of ^111^In. This will obviously reduce the statistics but improve the specificity [28, 29].

Images of the chest, abdomen and pelvis should be obtained for at least 100,000 counts but images over the peripheral skeleton may be acquired for time. As described above for ^99m^Tc, time presets decay-corrected can be used (see Table 1), although they are less relevant than for ^99m^Tc due to the long half-life of ^111^In. As ^111^In-WBC scintigraphy is preferable in low-grade infection, delayed and late imaging is usually sufficient and is not impaired by elution of the radiolabel from WBC. In abdominal infections or IBD, early imaging may be useful to differentiate between mucosal uptake that will decrease with time with intraluminal transit, sub-mucosal uptake that remains stable, and abscess that will show increasing uptake between 3 and 4 h and 20–24 h p.i. Given the low statistics due to the limitations in the injected activity for dosimetry reasons, SPECT or SPECT/CT may have to be performed with prolonged acquisition times. The very low background activity will nevertheless allow high contrast with such acquisition settings, even with low statistics.

When using ^111^In-labeled WBC for IBD evaluation, stool collection and counting at early and late time point may indicate the presence of radiolabelled cells migrating from infected mucosa to the lumen, an indirect sign of inflamed bowel. Planar acquisition of the pelvis in outlet views (namely, with the patient sitting above the gamma camera) or SPECT/CT may discriminate better between rectal and bladder activity in case of suspected recto-sigmoidal extent of IBD [32, 33].

#### Acquisition protocol for bone marrow scintigraphy (for bone and prosthetic joint infection)

Bone marrow scintigraphy is usually performed in doubtful WBC scans for bone and prosthetic joint infections. These are performed at the end of the WBC/mAb study (same day of the 24 h image or in the next few days) by injecting i.v. about 185 MBq (5 mCi) of ^99m^Tc-colloids (colloids of greater than 500 nm are recommended) and acquiring images of the region of interest after a minimum of 20–30 min and a maximum time of 6 h p.i. Images should be performed of the same views as those acquired with WBC/mAbs although the acquisition time should be limited to 300 s (if performed immediately at the end of WBC/mAbs scan) or 500,000 counts (if performed one or more days after WBC/mAbs scan). Another option in patients with violated bone is to perform ^99m^Tc-colloid marrow imaging simultaneously with WBC scans if labelling is done with ^111^In-oxine/tropolone. In this case, during ^111^In-WBC imaging only the upper peak of the ^111^In emissions should be used [28].

### Interpretation criteria for WBC and anti-G-mAbs

Given a correct acquisition protocol, images must be correctly displayed on the screen to allow their correct interpretation. Most work stations are pre-set to display multiple images in % of maximum counts/pixel. This display does not allow the reader to evaluate modifications of activity with time in the regions of interest. Furthermore, this kind of display needs the reader to adjust the intensity scale of each image to make bone marrow activity comparable and therefore introduces an operator bias [1, 69, 70].

All images acquired at different time points must be displayed with the same intensity scale in absolute counts, when they have been acquired with a time-decay corrected protocol. Any adjustment of the intensity scale must be applied to all images together, thus avoiding any operator bias.

Further interpretation of labeled WBC/mAb scintigraphy requires knowledge of the normal variants and pitfalls of these studies. The normal biodistribution of the radiopharmaceuticals has been summarized in Table 2, with uptake ranging from – (no activity) to +++ (the most intense activity). Diagnosis of infection is made by comparing delayed (3–4 h) and late images (20–24 h).

**Table 2:** Normal biodistribution of radiopharmaceuticals

Location	^99m^Tc-WBC early (30′-1 h)	^99m^Tc-WBC delayed (3-4 h)	^99m^Tc-WBC late (20-24 h)	^111^In-WBC (3 h/24 h)	^99m^Tc-mAbs (3 h/24 h)
Blood/heart	+++	+	–	±	±
Lung	+	–	–	−/−	−/−
Liver	++	++	++	++/++	+++/+++
Spleen	+++	+++	+++	+++/+++	+/++
Kidneys	+	+	+	−/−	+/+
Bladder	–	+	+	−/−	+/+
Bowel	–	+	++	−/−^a^	−/−
Bone marrow	+	++	++	++/++	+++/+++

^a^ With ^111^In WBC any bowel activity is abnormal; swallowed neutrophils from upper and lower respiratory tract could be considered in some cases of mild bowel activity

Images are then classified as (a) negative, when there is no uptake or there is a clear decrease of activity from delayed to late images in the regions of interest, (b) positive, when a clear increase is seen with time of uptake intensity or size in the regions of interest, and (c) equivocal, in all other situations. For OM in general, the early images at 30 min p.i. should be considered as a “vascular/bone marrow phase”. It is useful to compare it with the distribution of bone marrow. It must therefore be seen as a qualitative image and not considered for quantitative analysis compared to delayed and late images [1, 69, 70].

There might be several reasons for defining a set of images as “doubtful”. These are: similar or slightly decreasing uptake with time in the regions of interest (intensity or size); increase of bone marrow with time that does not allow to easily evaluate suspected regions; slight increase in size over time without an increase in intensity of uptake; slight increase in size and/or intensity over time in the region of interest but with a similar increase over time of bone marrow activity; activity in region of interest that remains unmodified with time (in size and/or intensity) with bone marrow activity that increases or decreases over time.

In all doubtful cases, following visual assessment, a semi-quantitative evaluation can be performed for the differential diagnosis of infection from non-specific uptake [69, 71, 76, 77]. Regions of interest (ROIs) are drawn over the area of interest (usually with the highest uptake) and copied to a presumed normal reference tissue; contra-lateral bone is the best when available [69]. The mean counts per pixel in these ROIs are recorded and used to calculate the lesion-to-reference (L/R) ratio in both the delayed and late images (L/R_delayed_ and L/R_late_, respectively). When the L/R ratio increases with time (L/R_late_ > L/R_delayed_) by at least 10%, the study can be considered indicative of infection; when the L/R_late_ is similar or decreases slightly with respect to L/R_delayed_ the examination can be classified as equivocal; and when L/R_late_ is significantly decreased compared to L/R_delayed_ (L/R_late_ < L/R_delayed_) the examination can be classified as negative for infection [69, 76].

If SPECT/CT is performed the delineation of the site of increased radiopharmaceutical uptake may be calculated by a 50% isocontour on a single transaxial slice that shows the site with the highest uptake (the lesion) and the reference normal tissue. The same criteria as described above can be used for imaging classification.

#### Prosthetic joint infection (PJI) and peripheral bone osteomyelitis (OM)

The above described criteria should be used for the diagnosis of peri-prosthetic joint infection and osteomyelitis.

Since labeled WBC and anti-G-mAb scintigraphy have a very high sensitivity, a negative scan (when there is no increased uptake of WBC with time) is sufficient to exclude the presence of bone infection.

In case of equivocal qualitative analysis, a semi-quantitative analysis should be performed, and only in case of equivocal quantitation (less than 10% increase of T/B over time) bone marrow imaging must be used. Colloids, anti-G-mAbs and labeled WBC accumulate in healthy and displaced bone marrow, whereas, in infection sites, colloids do not accumulate.

The WBC or anti-G-mAb scintigraphy is positive for osteomyelitis when there is activity on the primary scan without corresponding activity on the bone marrow image. When any other pattern is present, the study is negative for infection [13, 28, 69].

There are sufficient evidence based data to support the use of both WBC and anti-G-mAb for the diagnosis of PJI and OM as well as for FDG-PET/CT, only for OM and not for PJI.

One particular mention deserves the diagnosis of sternal wound infections (SWI) after thoracotomy and the criteria for the differential diagnosis between superficial infection (that requires medical treatment) and deep infection (that may require surgical treatment). In particular, if planar images at 3 h and 20 h are considered, a linear homogeneous uptake that decreases with time or remains stable over time can be considered as no infection or superficial infection. An inhomogeneous uptake with areas of increased uptake over time can be considered as an infection, most likely a deep infection and rarely a superficial infection. SPECT/CT acquisition can also be of help for the correct evaluation of the extent of infection.

#### Diabetic foot (DF)

In diabetic foot, to identify a Charcot foot an additional scan with colloids for bone marrow imaging is mandatory to differentiate between expanded bone marrow (a common finding after many surgical interventions to the bone as well as in Charcot foot) and OM [13, 14, 28]. There are sufficient evidence based data to support the use of both WBC and anti-G-mAb for the diagnosis of DF as well as for FDG-PET/CT.

#### Inflammatory bowel diseases (IBD)

Radiolabelled autologous WBC are a second-line imaging technique in IBD diagnosis used in case radiological and/or endoscopic exams are inconclusive [31]. The absence of abdominal uptake of WBC excludes the presence of IBD while the presence of pathological uptake can be due to IBD or to other disorders. WBC scintigraphy is positive when there is an increase of uptake of WBC in delay images (2–3 h p.i). Differential diagnosis between UC and CD is possible according to the pattern and localisation of the WBC uptake: if the uptake is in the ileo-cecal area, in the small bowel and there is a patchy distribution of radioactivity, it is more indicative of CD. Otherwise, when the leukocytes uptake is focused in left colon up to the rectum, or diffuse uptake in the colon, it is more characteristic of UC. If there is a colon uptake alone, it is not possible to establish a certain differential diagnosis. Major indications for WBC scan in IBD patients are: the early assessment of recurrences after surgery and the differential diagnosis between inflammatory and fibrotic strictures, the first being positive and the second negative. There are no sufficient evidence based data to support the use of anti-G-mAb scan in IBD.

#### Vascular graft prosthesis infection (VGI)

Radionuclide imaging studies are usually complementary to radiologic imaging, and limited to those patients with equivocal conventional imaging. ^99m^Tc-HMPAO-WBC scintigraphy has shown very high sensitivity and specificity in the evaluation of vascular prosthesis infection based on the presence of pathologic accumulation of labeled WBC in the site of infection as early as 2–3 h post-injection [49]. As previously described, early dynamic images and oblique images are useful integration to antero-posterior whole body scans, but SPECT or SPECT/CT images are often mandatory to discriminate the exact location of any suspicious uptake seen in planar images.

There are sufficient evidence based data to support the use of both WBC and of FDG-PET/CT in VGI.

#### Infective endocarditis (IE)

Nuclear medicine imaging techniques may be of great value in cases of undetermined echocardiographic findings (i.e., artifacts deriving from mechanical prosthesis) in the suspicion of an infective endocarditis and in detecting septic embolism (ref EHJ). Scintigraphy with labeled WBC should be performed always with SPECT/CT acquisition that enable obtaining high resolution images of the thorax. When there is a progressive accumulation of WBC in the cardiac region (for example, valve leaflets) the scintigraphy is positive. WBC scintigraphy is negative when there is no pathological uptake of WBC in the cardiac region while it is equivocal when there is a stable or decreasing accumulation of labeled cells. Since two SPECT acquisitions must be performed (usually at 5 h and 20 h post-injection) the acquisition time of each examination can be calculated according to isotope decay, in order to have the same statistics in both images and avoid operator bias in the interpretation of findings. Total body scans at 3–4 h and 21 h post-injection (before and after SPECT acquisitions, respectively) should also be performed to detect possible septic embolisms [2, 46, 47]. For prosthetic valves, it is important to analyze both CT-attenuated and non-attenuated (NAC) images since the positivity at NAC excludes a false positive finding due to metallic artifacts.

There are sufficient evidence based data to support the use of both WBC and FDG-PET/CT for IE but not for the use anti-G-mAb.

#### Fever of unknown origin (FUO)

Labeled WBC scan is usually adopted as second-line diagnostic investigations in the diagnosis of FUO, even if their overall performance is very satisfactory. The pre-test probability of infection is important and in case of low pre-test probability of infection (based on routine blood tests) an FDG-PET/CT scan is the preferred choice. In case of high white blood cell counts, ESR or CRP values a WBC scan can be performed as first imaging modality. Indeed, due to high sensitivity and specificity of WBC scintigraphy (60–85% and 78–94%, for ^111^In-oxine; 96 and 92% for ^99m^Tc-HMPAO), this can be considered the procedure of choice in patients with FUO with high probability of infection [78–80]. By contrast, there are no sufficient evidence based data to support the use of anti-G-mAb scan in FUO. Image interpretation criteria depends on the region of pathologic uptake of the labeled WBC, according to that mentioned above.

#### Pulmonary infections (PI)

There are too few reports (mainly case reports or AIDS patients) to define a specific imaging protocol for pulmonary infections. Planar antero-posterior images are generally sufficient, given the considerations and pitfalls mentioned above.

#### Central nervous system infections (CNS)

Scintigraphy with radiolabelled WBC has shown to be very sensitive and specific in CNS infections and other head and neck infections such as malignant otitis, sinusitis, dermal filler infections and skull osteomyelitis [55–63, 71]. Due to high physiological brain uptake of FDG, to date, WBC scintigraphy is the only and most accurate nuclear medicine technique for H&N infections. Planar antero-posterior images and latero-lateral images are often sufficient but SPECT and SPECT/CT acquisitions can be mandatory in some low grade infections [71].

Image acquisition protocols and interpretation criteria are summarized in Tables 3, 4 and 5.

**Table 3:** Summary of acquisition/interpretation protocols of musculoskeletal infections

Examination	Acquisition protocol	Image interpretation	Pitfalls
Osteomuscular (osteomyelitis, and prosthetic joint infection)	Labeled WBC and ^99m^Tc-mAb-anti-granulocytes: 30 min p.i. planar images for QC and ROI - 3-4 h p.i. total-body and planar images ROI 20-24 h p.i.: planar and SPECT(/CT) ROI - ^99m^Tc-sulfur colloid 20–360 min p.i. planar images ROI	Qualitative analysis: Infective lesions show an uptake that arise over time while aseptic flogistic lesions show a decreasing uptake over time. In doubtful cases it is necessary to compare the images with ^99m^Tc-sulfur colloids: infective lesion shows a mismatch pattern of radiopharmaceutical uptake. Semi-quantitative analysis: Calculate the target/background ratio drawing ROI on the regions that show an increased uptake over time (T) and on contralateral bone marrow (B) FP results can be obtained if the scintigraphy is performed within 3–4 months after surgery	Assess timing after surgical procedure. Assess the presence of artifacts related to the attenuation over-correction in patients with metallic devices
Discitis, spondylitis and spondylodiscitis	^99m^Tc-HDP/MDP: i.v. injection during dynamic acquisition 4 h p.i. total-body, planar and SPECT(/CT) images ROI ^67^Ga-citrate 4 h p.i. total body, planar and SPECT(/CT) images ROI 24 h p.i. planar images ROI 48 h p.i. planar and SPECT(/CT) images ROI, if necessary [^18^F]FDG PET/CT 1 h p.i. PET/CT	Clinical history (timing from surgical procedure, and type of metallic implant, etc). Anatomic location of tracer uptake. ^99m^Tc-HDP/MDP + ^67^Ga-citrate: Infective lesions show usually greater uptake of ^67^Ga in comparison to ^99m^Tc-HDP/MDP while an aseptic process (arthritis, fracture etc) shows greater uptake of ^99m^Tc-HDP/MDP than that of ^67^Ga. [^18^F]FDG PET/CT: Infective lesions show greater uptake of [^18^F]FDG than healthy vertebral body	Assess timing after surgical procedure. Assess the presence of artifacts related to the attenuation over-correction in patients with metallic devices
Sternal infections	Labeled WBC and ^99m^Tc-mAb-anti-granulocytes: 30 min p.i. Planar images for QC and ROI 3–4 h p.i. total-body, planar and SPECT(/CT) images ROI. 20–24 h p.i. planar and SPECT(/CT) images ROI	Qualitative analysis: an increased uptake with time of sternum and/or mediastinum is indicative of infection. Semi-quantitative analysis: *Pattern I*: Presence of deep infection. Widespread and intense uptake of the sternum, greater than liver uptake, after 3–4 and 20–24 h. *Pattern II*: Presence of superficial infection. Moderate increase or irregular uptake of sternum that does not change or decrease between 3 and 4 and 20–24 h. *Pattern III*: Absence of infection. Medium intensity uptake with uniform distribution of the sternum, abnormal uptake (“cold” areas) on the midline or cold areas with focal distribution in the midline	Medicate the wound before scintigraphic acquisition to avoid FP results. FP results: missed clearing of wound before scintigraphic acquisition
Diabetic foot	Labeled WBC and ^99m^Tc-mAb-anti-granulocytes: 30 min p.i. planar images for QC and ROI 3–4 h p.i. total-body and planar images ROI 20–24 h p.i. planar and SPECT(/CT) images ROI ^99m^Tc-sulfur colloids 20–360 min p.i. planar images	Semi-quantitative analysis: Infective lesions show an uptake that rises over time while aseptic flogistic lesions show a decreasing uptake over time. In doubtful cases it is necessary to compare the images with ^99m^Tc-sulfur colloids infective lesion to check for a mismatch pattern of radiopharmaceuticals uptake	Clean the wound before scintigraphy acquisition to avoid FP. Assess the presence of lesions of size lower than spatial resolution of method

*QC* quality control, *ROI* region of interest

**Table 4:** Summary of acquisition/interpretation protocols of soft tissues infections

Examination	Acquisition protocol	Image interpretation	Pitfalls
Central nervous system infections	Labeled WBC: 30 min p.i. planar images for QC and ROI 3–4 h p.i. total-body and planar images ROI 20–24 h p.i. planar and SPECT(/CT) images ROI	Semi-quantitative analysis: Infective lesions show usually an uptake equal to +++/++++ while aseptic lesions show no uptake or equal to +/++	
Infective endocarditis	Labeled WBC: 30 min p.i. planar images for QC and ROI 3–4 h p.i. total-body, planar and SPECT(/CT) images ROI 20–24 h p.i. planar and SPECT(/CT) images ROI	Qualitative analysis: analysis of cardiac region (valve plans) and other regions (CNS, spleen and axial skeleton, lung) Anatomic location of WBC uptake. Correlation with flogosis markers and echocardiography.	Assess the presence of artifacts related to the attenuation overcorrection for the presence of surgical implants (mechanical valve prostheses and CIED). FN results: fungal endocarditis FP results: atherosclerotic plaques, sarcoidosis, tumor, vasculitis etc
Post surgical infections (dermal filler infections and abscesses)	Labeled WBC: 30 min p.i. Planar images for QC and ROI 3–4 h p.i. total-body, planar and SPECT(/CT) images ROI 20–24 h p.i. planar and SPECT(/CT) images ROI	Clinical history (date and sort of surgical procedure). Anatomic location of WBC uptake. Correlation with flogosis markers	Assess the presence of artifacts related to the attenuation overcorrection in patients with metallic devices
Pulmonary infections	Labeled WBC: 30 min p.i. Planar images for QC and ROI 3–4 h p.i. total-body planar and SPECT(/CT) images ROI 20–24 h p.i. planar and SPECT(/CT) images ROI	Focal pulmonary uptake that arise over time is usually associated with bacterial pneumonia. Diffuse pulmonary activity on images obtained 4 h p.i. can be due to opportunistic infections, radiation pneumonitis, pulmonary drug toxicity and adult respiratory distress syndrome	FP results: cystic fibrosis, faulty labelling or reinjection

*QC* quality control, *ROI* region of interest

**Table 5:** Summary of acquisition/interpretation protocols of others infections

Examination	Acquisition protocol	Image interpretation	Pitfalls
Fever of unknown origin	Labeled WBC and ^99m^Tc-mAb-anti-granulocytes: 30 min p.i. planar images for QC and ROI 3–4 h p.i. total-body and planar images ROI 20–24 h p.i. planar and SPECT(/CT) images ROI	Evaluation of all regions of increased uptake having regard to the possible differential diagnosis (inflammation, cancer)	Assess the presence of artifacts related to the attenuation overcorrection in patients with metallic devices or prostheses
Inflammatory bowel diseases	Labeled WBC and ^99m^Tc-mAb-anti-granulocytes: 30 min-1 h p.i. planar images for QC and ROI 2–2.5 h p.i. planar and SPECT(/CT) images ROI planar image of the chest (in patients with suspected oesophageal localization of disease). 24 h p.i. planar images ROI for ^99m^Tc mAb or for abscesses and fistulae	Abscesses and fistulae can appear only at late images. Better to use SPECT/CT in case of doubt. FP results can be obtained in tumor. Aspecific uptake in colitis, bleeding and diabetic patients	Starting from 3 h p.i. due to aspecific accumulation of secondary hydrophilic complexes of Tc in caecum and ascending colon after 2 h p.i.
Vascular prosthesis	Labeled WBC and ^99m^Tc-mAb-anti-granulocytes: i.v. injection during dynamic acquisition (one image every 5–10 s for 3–5 min) 30 min p.i. planar images for QC and ROI 3–4 h p.i. total-body, planar and SPECT(/CT) images ROI 20–24 h p.i. recommended if the chest is the ROI	Clinical history (date and sort of vascular prosthesis implant, etc). Anatomic location of WBC uptake (in vascular prosthesis wall, at the site of surgical clips and/or in soft tissues)	Assess the presence of artifacts related to the attenuation overcorrection in patients with metallic clips. FN results: chronic flogistic process with low recruitment of WBC

*QC* quality control, *ROI* region of interest

### Additional strategies that may improve accuracy

If SPECT/CT is not available SPECT WBC or anti-G-mAb images can be co-registered with separately acquired CT or MRI images (usually transaxial slides) for a more accurate localization of the pathological uptake, but attention should be paid to the fact that thickness of SPECT and CT slides is different.

New software is also available for SUV calculation on SPECT images.

### Physiologic biodistribution, pitfalls and artifacts

After in vivo administration of radiolabelled WBC, the cells normally show a transitory migration to the lungs (margination) and then accumulate in the spleen and, to a lesser extent, in the liver and bone marrow, with a maximum uptake at 2–4 h p.i.. Labeled neutrophils accumulate preferentially in the liver and eosinophils preferentially into the spleen. During the following hours, labeled cells migrate from spleen and bone morrow to infected tissues. This is the reason why bone marrow activity decreases with time and infection shows an increase of activity with time. Although pulmonary uptake of labeled WBCs is physiologic during the first few hours after injection, at 4 and 24 h p.i. such lung activity is abnormal. Focal segmental or lobar pulmonary uptake is also associated as a rule with infection. Non-segmental focal pulmonary uptake in early images, however, is usually caused by technical problems during labelling or reinfusion and is generally not associated with infection.

Cell migration is largely influenced by the vascularization on the infected region. In spondylodiscitis there a reduced blood flow due to oedema that compresses small capillaries and makes it difficult for granulocytes to accumulate in the region. Similarly, in diabetic foot infection a poor vasculature of distal toes has been described, thus migration of leukocytes can be impaired in soft tissue infections but still present when bone is infected. This different trend between soft tissue infection and osteomyelitis has been reported only for diabetic foot infections and can allow differentiation of soft tissue infections from osteomyelitis but not soft tissue infections from sterile inflammation [14].

^111^In-labeled WBC do not accumulate in the normal bowel. Intestinal activity is always abnormal and should be seen in antibiotic-associated colitis, pseudomembranous colitis, infectious colitis, IBD, ischemic colitis or gastrointestinal bleeding.

WBC do not usually accumulate in healing surgical wounds and their presence in these sites indicates a soft-tissue infection. When the wound is very close to the bone surface (as in feet, tibia, skull, etc.) it might be difficult to discriminate between soft tissue involvement alone or bone involvement too. Since OM is always much more clinically relevant than a soft tissue infection, a differential diagnosis must be made and SPECT/CT acquisitions may help in this evaluation.

There are, however, certain exceptions to the rule. Granulating wounds that heal by secondary intention can appear as areas of intense uptake on WBC images even in the absence of infection. Examples include stomas (e.g. tracheostomy, ileostomy, feeding gastrostomy, etc.) and skin grafts. Vascular access lines, dialysis catheters and even lumbar punctures can all produce false-positive results and therefore the importance of knowledge of the clinical history.

A possible cause of non-infective accumulation of WBC in the bone is the Paget disease.

With complete and fragmented mAbs some differences in physiological uptake should always be taken into consideration: complete ^99m^Tc-anti-NCA-95 antibody scans show intense uptake in bone marrow, spleen > liver already 1–4 h p.i., whereas both kidneys are shown only slightly. Non-specific bowel activity is regularly seen after 20–24 h p.i. due to the beginning of radiolabel instability.

With ^99m^Tc-anti-NCA-90 (Fab’) antibodies, bone marrow is shown in a much lower degree as compared with complete antibodies, the uptake of liver > spleen, and very intense uptake is seen in the kidneys due to predominantly renal excretion of ^99m^Tc-anti-NCA-90 (Fab’) and non-specific bowel activity is already seen 4–6 h p.i. due to enzymatic liver degradation of the compound.

Monoclonal antibodies allow rapid and safe delineation of inflammatory foci by efficient accumulation on the surface of chemotactic activated granulocytes. The decrease of molecular size increases the background clearance together with a significant reduction of non-specific accumulation in other organs hampering image interpretation. Antibodies bind to granulocytes with high affinity (with K_d_ in the nanomolar range) being specifically involved in the process of infection without undesirable expression of targets in non-infected tissues.

Furthermore, it is generally accepted that anti-G-mAbs localize in infectious foci by two pathways: (a) in-vivo targeting of chemotactically activated granulocytes and (b) non-specific extravasation due to the locally enhanced vascular permeability, the later allowing delayed targeting at 10–20% ID.

Monoclonal antibodies visualize infectious foci in patients with a sensitivity between 80 and 90%, are very useful in the evaluation of bone prosthetic infections (and in this case a combination with 3-phase bone scan is highly recommended) as well as of soft tissue infections such as vascular graft infection, prosthetic heart valve infection and inflammatory bowel disease [80–83], although these agents appear to be somewhat less accurate than labeled leukocytes. Pulmonary infections—with the exception of lung abscesses—are not easily visualized. Peripheral bone infections can be adequately visualized, but the sensitivity decreases if the focus is located closer to the spine [84].

The major disadvantages of the murine monoclonal antibodies are that they are registered in Europe only for osteomyelitis diagnosis and that they may induce formation of human anti-mouse antibodies (HAMA), which can result in altered biodistribution of subsequent injections [84]. Colorimetric kit tests for rapid measurements of HAMA, however, are now commercially available and very easy to use giving a result in less than 5 min.

#### Report of scintigraphic findings

The final report of the study should be divided into five parts: identification, clinical question, procedure description, report text and conclusions.

- **Identification**: this includes the patient identification, the institution where the scintigraphy was performed, the date of scintigraphy, the type of scintigraphy, the name of radiopharmaceutical and the activity administered to patient (MBq), and any other specification required by national regulations, e.g. the name of the radiographer performing the study.
- **Clinical question:** this includes the clinical question and brief clinical history of the patient. Current treatment with antibiotics or other interfering drugs should be reported.
- **Procedure description:** this includes the description of instrumentation used, the administration method of the radiopharmaceutical and the acquisition protocol. The use of SOPs should be mentioned as well as the European Guidelines for labelling, image acquisition and interpretation.
- **Report text:** this includes the description of qualitative and/or semi-quantitative analysis. The qualitative analysis should describe the presence/absence of radiopharmaceutical *uptake*, the site and size of uptake, and the intensity of uptake, preferably supported by semi-quantitative data. The possible presence of factors that may have limited the sensitivity and specificity of the study, such as the presence of motion artifacts, should be described.
- **Conclusions:** This is the clear and conclusive answer to the clinical question. It can also suggest other diagnostic procedures to be performed to confirm or exclude the diagnosis made. The anatomical structures involved, as well as the presence, extent and the intensity of the infectious process have to be specified. At the end of the conclusions the name and surname of the Nuclear Medicine Physician reporting the study and of the technician performing the scan have to be clearly stated.
